# A novel MSMB-related microprotein in the postovulatory egg coats of marsupials

**DOI:** 10.1186/1471-2148-11-373

**Published:** 2011-12-30

**Authors:** Stephen Frankenberg, Jane Fenelon, Bonnie Dopheide, Geoff Shaw, Marilyn B Renfree

**Affiliations:** 1 ARC Centre of Excellence for Kangaroo Genomics and Department of Zoology, University of Melbourne, Parkville, Victoria, Australia

## Abstract

**Background:**

Early marsupial conceptuses differ markedly from those of eutherian mammals, especially during cleavage and early blastocyst stages of development. Additionally, in marsupials the zona pellucida is surrounded by two acellular layers, the mucoid coat and shell, which are formed from secretions from the reproductive tract.

**Results:**

We report the identification of a novel postovulatory coat component in marsupials, which we call uterinesecreted microprotein (USM). USM belongs to a family of disulfide-rich microproteins of unconfirmed function that is found throughout deuterostomes and in some protostomes, and includes β-microseminoprotein (MSMB) and prostate-associated microseminoprotein (MSMP). We describe the evolution of this family in detail, including USM-related sequences in other vertebrates. The orthologue of *USM *in the tammar wallaby, *USM1*, is expressed by the endometrium with a dynamic temporal profile, possibly under the control of progesterone.

**Conclusions:**

USM appears to have evolved in a mammalian ancestor specifically as a component of the postovulatory coats. By analogy with the known properties of MSMB, it may have roles in regulating sperm motility/survival or in the immune system. However, its C-terminal domain is greatly truncated compared with MSMB, suggesting a divergent function.

## Background

Marsupial conceptuses are surrounded by three extracellular investments (reviewed [1]). The innermost layer, the zona pellucida, is deposited during oogenesis and occurs in all mammals. After ovulation and fertilisation, it becomes surrounded by a thick, translucent layer mucoid coat that is deposited during passage through the oviduct and traps non-fertilising sperm. By the time the conceptus arrives in the uterus, the mucoid coat has become surrounded by a thin, dense, shell coat derived mainly from secretions in the utero-tubal junction and the uterus [2-4]. During the period we define as "preliminary blastocyst expansion", the mucoid coat narrows as it becomes compressed between the expanding zona pellucida and the outer shell coat. During "secondary expansion", the shell coat itself expands from an initial diameter of about 200-300 μm up to ~17 mm, increasing its volume dramatically from 0.001 mm^3 ^to > 0.250 mm^3 ^[5]. The shell coat finally ruptures approximately two-thirds of the way through pregnancy, or 3-8 days before birth [6], under the influence of proteases secreted by the endometrium [7], after which attachment occurs.

A previous study [2] made substantial progress in identifying components of the postovulatory coats of the brushtail possum (*Trichosurus vulpecula*) and the stripe-faced dunnart (*Sminthopsis macroura*). The authors isolated individual protein components by electrophoresis and sequenced their N-terminal regions. The short sequences obtained (12-15 residues) for twelve excised protein bands (seven from possum and five from dunnart) could not initially be identified due to insufficient bioinformatic resources for these species at the time. Since that study, one band was identified as similar to τ-crystallin/enolase 1 and termed CP4 (coat protein 4) [8].

Genomes have now been sequenced from two marsupials - the South American grey short-tailed opossum (*Monodelphis domestica*) [9], and more recently the Australian tammar wallaby (*Macropus eugenii*) (in press). With these new resources at hand, we re-examined the published protein sequences of Casey et al. [2] and identified one of them from the brushtail possum. We show that the gene encoding this protein, which we call uterinesecreted microprotein (*USM*) is a paralogue of *MSMB *(β-microseminoprotein; also called PSP94, β-inhibin and IgBF). MSMB is a disulfide-rich, low molecular weight protein that is a major component of seminal fluid and is strongly expressed in the prostate gland as well as in other tissues, especially of the reproductive system and in mucosal membranes. Its specific function is not known, but it may have roles in inhibition of sperm motility [10,11], suppression of immune response against allogeneic sperm [12], toxin defence [13,14], pituitary-gonadal axis signalling [15-17] and suppression of prostate tumorigenesis [18-22]. Very little is known of MSMP, which is similar to MSMB but more highly conserved among species, apart from its expression in a prostate cancer cell line [23]. USM is similar to both MSMB and MSMP in its conserved sequence of disulfide bond-forming cysteine residues, but most of the region homologous to the C-terminal domain of MSMB is absent. In this study, we examine the evolution of USM/MSMB/MSMP-related microproteins in vertebrates. We discuss how, as a component of the marsupial postovulatory coats, USM could provide important clues for elucidating the roles of MSMB and other related proteins, with possible applications in prostate cancer, immunity and fertility control.

## Results & Discussion

### Identification of postovulatory coat proteins

**Figure 1:**
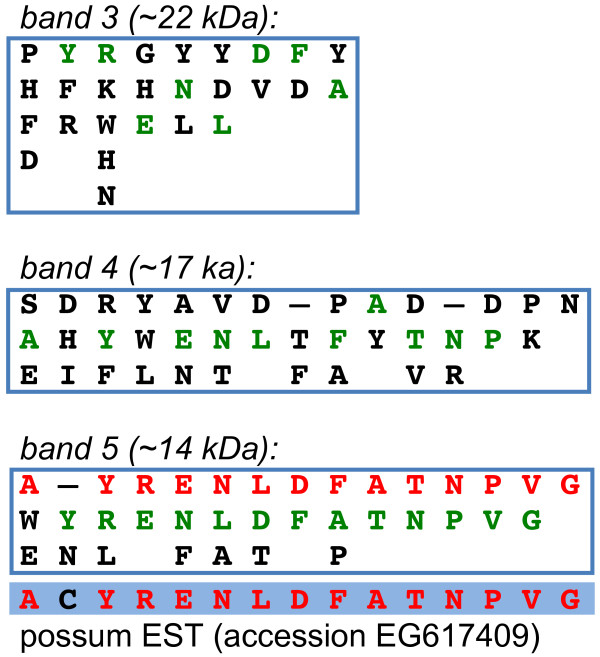
**Identification of a brushtail possum coat protein component from published sequences**. Published sequences (boxed) of electrophoresed protein bands from possum postovulatory coats [2] are aligned with a translation of brushtail possum expressed sequence tag (EST), GenBank accession EG617409, highlighted in blue. Aligned residues between the translated EST and the major sequence of band 5 are shown in red. Additional residues within the sub-sequences of bands 3-5 that also match the EST are shown in green. In bands 3 and 5, these are one residue out of phase. The approximate molecular weights indicated are those estimated by Casey *et al*. [2].

Protein sequences from Casey et al [2] were used to search GenBank databases using the tBLASTn algorithm with low-stringency search parameters. The alignments of sequences from Casey et al [2] with an expressed sequence tag [GenBank accession EG617409] derived from the reproductive tract of the brushtail possum is shown in Figure 1. The major sequence from Band 5 (14 kDa) matched closely the translated possum EST, while minor sequences from Bands 3 (22 kDa), 4 (17 kDa) and 5 also showed identity. We named this protein uterine secreted microprotein (USM).

**Figure 2:**
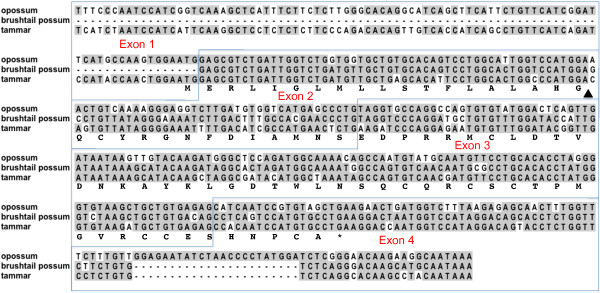
**Sequence and translation of tammar *USM1 *and opossum *USM *aligned with brushtail possum EST**. Note that the "first exon" in the brushtail possum EST is excluded from this alignment, as it appears to be an artefact (see text). Sequences extend from the transcription start site, determined for tammar *USM1 *by 5' RACE, to the predicted polyadenylation signal. The signal peptide cleavage site is indicated by an arrowhead.

The translated sequence of the possum EST was used to identify exons in the tammar wallaby (*Macropus eugenii*) whole genome shotgun (WGS) database, which revealed an apparent four-exon structure with an open reading frame spanning Exons 2-4 (Figure 2). A second, more divergent homologue was also identified bioinformatically. We refer to these genes respectively as tammar *USM1 *and *USM2*. Conserved exons in the opossum genome were identified as a homologue of *USM*.

**Figure 3:**
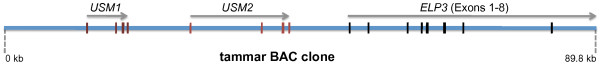
**Structure of sequenced tammar BAC clone**. The positions of exons of *USM1, USM2 *and *EPL3 *within a single 89.8 kDa contig sequenced from a tammar genomic BAC clone are shown, drawn to scale. Arrows denote the orientation (5'-to-3') of the sense strand of coding regions.

Exon 1 of tammar *USM1 *was determined by 5' RACE and differed from that of the brushtail possum EST. In the opossum genome, Exons 2-4 of the brushtail possum EST map to Chromosome 1 whereas the "first exon" maps to Chromosome 8, immediately upstream of the third exon of another gene, *WASH1*. To resolve this discrepancy and to characterise fully the genomic locus of tammar *USM1*, we isolated and sequenced a tammar genomic BAC clone containing the gene. In a single assembled 89.8-kb contig of BAC sequence [GenBank accession JN251945], no sequence matching the "first exon" of the brushtail possum EST was present in the 17.1 kb upstream of Exon 2, however Exon 1 as identified by 5' RACE was located upstream of Exon 2, as expected. *USM2 *was located downstream of *USM1 *in the BAC sequence and in the same orientation. *USM2 *also contains exons homologous to Exons 1-4 (Figure 3). We conclude that the "first exon" of the brushtail possum EST represents an anomaly or an artefact of cDNA library construction. Downstream of *USM2 *and in the same orientation as *USM1 *and *USM2 *in the BAC sequence, we identified the first 8 exons of *ELP3 *(Figure 3), which also flanks *USM *in the opossum genome. This confirmed that opossum *USM *is orthologous to the tammar *USM1/USM2 *cluster. However, unlike in the tammar, no duplicate of *USM *was found at this locus in the opossum.

A signal peptide cleavage site was predicted at the same position in all three species (Figure 2 and additional file 1: Protein_alignment.pdf), strongly indicative of a secretory protein and consistent with a role in the extracellular postovulatory coats. In the brushtail possum, the predicted cleavage site also immediately precedes the major sequence from band 5 (compare Figure 1 and additional file 1: Protein_alignment.pdf), which was obtained by N-terminal sequencing.

In eutherian sequence databases, the highest translated sequence identity with the *USM *genes was found in orthologues of *MSMB *and another related gene, *MSMP *(also called *PSMP*). *USM *is not an orthologue of either of these genes, however, as other genes corresponding respectively to orthologues of *MSMB *and *MSMP *were identified in both tammar and opossum genomes. Thus *USM *is a novel mammalian gene that is absent in the eutherian lineage.

**Figure 4:**
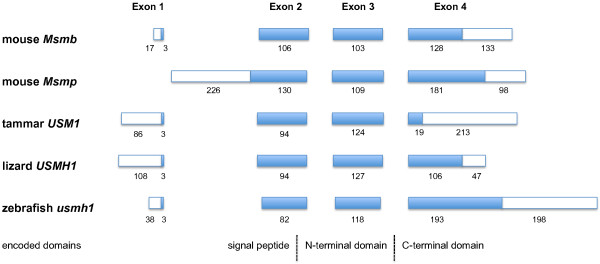
**Comparison of exon structure of selected *USM/MSMB/MSMP *family members**. Regions encoding the signal peptide and N- and C-terminal domains are indicated. Filled boxes represent coding regions. Numbers represent nucleotide lengths.

The four-exon structure of marsupial *USM *genes is similar to that of *MSMB*, including a predicted translation initiation codon in the three 3'-most nucleotides of Exon 1 (Figure 4). *USM *and *MSMB *both differ from *MSMP*, which is comprised of only three coding exons, homologous to *USM/MSMB *Exons 2-4. Thus for ease of comparison, the exons of *MSMP *are hereafter referred to according to their homology with *USM/MSMB *exons. The full coding region and splicing structure of tammar *USM1 *was confirmed by RT-PCR followed by cloning and sequencing (not shown).

### The *USM/MSMB/MSMP *gene family

To examine the evolution of the *MSMB/MSMP/USM *gene family, we performed low stringency tBLASTn searches of GenBank databases and identified numerous homologues in vertebrate genomes as well as in those of lower deuterostomes, including *Ciona *spp. (Urochordata), *Branchistoma lanceolatum *(Cephalochordata) and *Stronglyocentrotus purpuratus *(Echinodermata), and of protostomes, including those recently reported in the phyla Mollusca and Rotifera [24]. (See additional file 2: Sequence_sources.pdf for sources of all sequences used in this study.) Most of the identified genes were previously unreported. Alignment of a large number of translated sequences (not shown) suggested a complex pattern of evolution with rapid sequence changes and gene duplication events. As previously reported [23], *MSMP *showed the strongest conservation among vertebrates. Because of the large number of amino acid substitutions, the phylogenetic relationship between family members from distantly related species was not readily resolved by standard bootstrapping methods, with the exception of *MSMP*-like genes (not shown). Nevertheless, three broad sub-families appeared to be represented among vertebrates: *MSMP*-like, *MSMB*-like and *USM*-like(see additional file 3: Tree.pdf).

### Conserved synteny among *MSMB/MSMP/USM *family members

**Figure 5:**
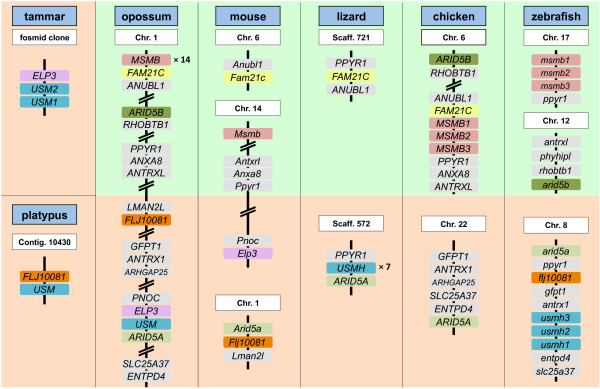
**Regions of conserved synteny among vertebrate genomes**. Only some genes, whose position within a syntenic group is informative, are shown. Thus a small number of intervening genes lie between some genes that are depicted as directly flanking for simplicity. Larger intergenic distances are depicted by parallel diagonal lines. Key genes mentioned in the text are highlighted in colour.

To clarify the relationships among *MSMB/MSMP/USM *family members, we examined their conservation of synteny with flanking genes. We focussed particularly on *MSMB*-like and *USM*-like genes as they showed the most sequence variability. The most informative syntenic groups are summarised in Figure 5.

In the opossum genome, *USM *flanks *ARID5A *while 14 tandem copies of *MSMB *flank *FAM21C *and *ANUBL1*. In the chicken, 3 tandem copies of *MSMB *also flank *FAM21C *and *ANUBL1*, which are located near *ARID5B *(a paralogue of *ARID5A*) on chromosome 6, whereas no *MSMB*/*USM*-like gene is located near *ARID5A *on chromosome 22. By contrast, *USM-*like genes lie close to *ARID5A *in the lizard genome and *arid5a *in the zebrafish genome. This suggests that the same duplication event that generated *ARID5A *and *ARID5B *also generated *USM*-like and *MSMB*-like genes, respectively. This duplication event can be traced to prior to the divergence of the teleost fish lineage (which has also undergone its own genome duplication event [25]) and is associated with the generation of other paralogous pairs (*ANTRX1/ANTRXL *and others not shown) that variably cluster with *ARID5A/ARID5B *in vertebrate genomes (Figure 5). Both of these paralogous syntenic groups variably contain a homologue of *PPYR1*, with some lineages (such as lizard and zebrafish) containing a homologue in both syntenic groups.

### *USM *homologues in other vertebrates

The presence of paralogous syntenic clusters conserved throughout vertebrates allowed orthologues of *USM *to be clearly identified. Among non-mammalian tetrapods, the most similar sequence to USM found was from the genome of the green anole lizard (*Anolis carolinensis*). Multiple copies of USM-related genes in the lizard flank *ARID5A*, as does opossum *USM *and a cluster of three *USM*-like genes in the zebrafish genome (Figure 5). These genes appear to be orthologous with respect to their origin, thus we refer to the lizard genes as *USMH1 *to *-7 *(*USM homologue 1 *to *7*) and the zebrafish genes as *usmh1 *to *-3 *(*USM homologue 1 *to *3*). They may not have equivalent function to *USM*, however, as the lizard and zebrafish genes have retained a complete Exon 4 encoding the C-terminal domain, in contrast to marsupial *USM *genes in which the open reading frame encoding this domain is greatly truncated. They also have four coding exons (Figure 4), which is supported by transcript evidence (see additional file 2: Sequence_sources.pdf). Published cDNA sequences from the Habu snake (*Trimeresurus flavoviridis*) [26] are similar to the lizard *USMH *genes. Like the lizard and zebrafish *USMH *genes, the snake genes are more similar to each other (not shown), suggesting that they also represent a lineage-specific expansion in copy number. These genes encode small serum proteins, SSP1-5, which appear to have a role in protection against the snake's own venom rather than in reproduction [13,14]. *USMH*-like sequences were also found in cDNAs derived from mixed tissues of the channel catfish (*Ictalurus punctatus*). According to the NCBI UniGene EST expression profiles, zebrafish *usmh *transcripts are found largely in the reproductive tract and consist mostly of *usmh2 *and *usmh3 *transcripts. Although it is possible that USMH proteins in other vertebrates also contribute to postovulatory coats, this appears unlikely due to their apparent additional expression in non-reproductive tract tissues. Furthermore, no specifically *USM*-like sequences were identified in the two sequenced avian genomes, chicken (*Gallus gallus*) and zebra finch (*Taeniopygia guttata*), in which conservation of postovulatory coat proteins might be expected. Thus it appears that a *USMH *gene evolved a novel role in the postovulatory coats of a common mammalian ancestor.

An apparent orthologue of *USM *is also present in the genome of the platypus (*Ornithorhynchus anatinus*), based on sequence similarity of Exons 1-3 and proximity to an orthologue of *Flj1008*, which also flanks *Arid5a *on mouse chromosome 1 (Figure 5). However platypus Exon 4 could not be identified either manually or using gene prediction software, thus it could not be determined whether it encodes the same C-terminal truncation as marsupial *USM*. Our failure to detect Exon 4 argues that it probably has a truncated open reading frame and therefore platypus *USM *is likely to be functionally equivalent to marsupial *USM *rather than other vertebrate *USMH *genes.

### MSMB paralogues in birds and marsupials

Our phylogenetic analysis of avian MSMB homologues revealed three distinct but previously unrecognised paralogous groups. The three chicken paralogues, which we term avian *MSMB1, MSMB2 *and *MSMB3*, flank each other on chromosome 6. Thus unlike in New World monkeys [27], in which multiple copies of *MSMB *appear to have arisen independently, avian *MSMB *paralogues are apparently conserved. Furthermore, each avian *MSMB *paralogue shows high conservation in its translated sequence with its respective orthologues. Previously characterised sequences from chicken and ostrich (*Struthio camelus*) designated as *MSMB *[15,28] correspond to *MSMB1*, while the gene currently annotated as *MSMB *by the NCBI "Gene" database http://www.ncbi.nlm.nih.gov/gene?term=msmb%20gallus corresponds to *MSMB2*. A partial transcript of *MSMB3 *from chicken [GenBank accession DT655693] is annotated as being derived from reproductive tract ("testis, ovary and oviduct"), while a full transcript from duck (*Anas platyrhynchos*) [GenBank accession HO188240] was derived from a screen for genes expressed in the epithelium of the magnum (part of the reproductive tract) and correlated with high egg hatchability [29].

Avian *MSMB1-3 *genes differ markedly from each other in their degree of conservation. Translated sequence identities of the *MSMB *paralogues of zebra finch (order Passeriformes) were compared with their respective orthologues from Anseriformes (duck) and/or Galliformes (chicken, duck and turkey), the latter two orders forming a monophyletic clade [30]. Conservation is notably higher among *MSMB3 *orthologues (81-82% amino acid identity) compared with *MSMB1 *(54-60%) and *MSMB2 *(53-56%) (see additional file 4: avian_MSMBs.pdf for table of sequence identities and similarities). The pattern was similar when comparing within Galliformes (chicken versus turkey): 83% amino acid identity for *MSMB1*, 89% for *MSMB2 *and 98% for *MSMB3*. Mouse *Msmb *is more similar to avian *MSMB2 *(32-36% amino acid identity) than either *MSMB1 *(25-28%) or *MSMB3 *(23-25%). These data suggest that avian *MSMB3 *has acquired a novel, specialised role in birds distinct from that of other vertebrate *MSMB *homologues. Considering the tissue source of the only two known transcripts, this role is likely to be related to reproduction. Ostrich MSMB1 was originally identified in the pituitary gland [15], supportive of a previously proposed role in the pituitary-gonadal axis [17,19,20], although this role was later refuted [31,32].

In the opossum, we identified fourteen paralogues of *MSMB*, which we termed *MSMB1 *to *-14*, flanking each other on chromosome 1 (Figure 5). Similarly in the tammar, we identified at least ten presumed *MSMB *paralogues, although not all exons could be identified and their synteny could not be confirmed. One tammar homologue, designated *MSMB1*, is very similar to opossum *MSMB1 *and presumably orthologous to it. *MSMB1 *from tammar and opossum are strongly divergent from the other *MSMB *homologues of both species and are significantly longer within Exon 3 (not shown). Thus only the *MSMB *paralogues that flank *MSMB1 *(presumably in tammar as well as opossum), but not *MSMB1 *itself, have undergone multiple duplications independently within each lineage.

**Figure 6:**
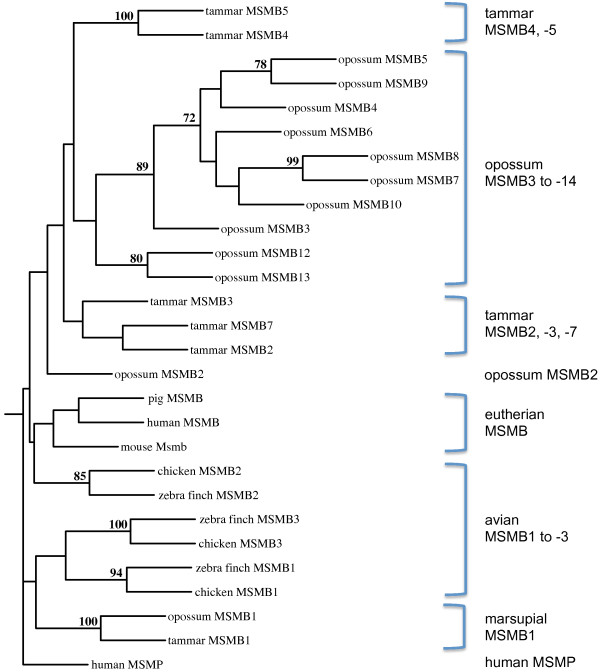
**Phylogenetic analysis of MSMB homologues in selected amniotes**. The phylogenetic tree was generated using translated sequences of Exons 3-4 from selected *MSMB *homologues (marsupial homologues with incomplete sequences were omitted), using the neighbor-joining method with 1000 replicates. Only bootstrap values of greater than 70% are shown at branching points. Human MSMP was used as an outgroup.

The above conclusions in birds and marsupials are supported by phylogenetic analysis (Figure 6). Significant (> 70%) bootstrap values were obtained supporting orthology of avian MSMB1, -2 and -3, respectively, between chicken and zebra finch, and of marsupial MSMB1 between opossum and tammar. No marsupial homologues other than MSMB1 showed significant bootstrap values between tammar and opossum, whereas homologues from the same species tended to cluster together within the phylogenetic tree, indicative of lineage-specific duplication events.

### *MSMB*-like genes in protostomes

Protostomal *MSMB*-like genes were identified mostly from the phylum Mollusca, including bivalves, gastropods and cephalopods, with one sequence from Rotifera. Additional identified transcript sequences were from a cDNA library derived from floral bulbs of Lewis' monkeyflower (*Mimulus lewisii*), a flowering plant. These are assumed to have arisen from contamination of the floral buds by a terrestrial gastropod (a slug or a snail) (H.D. Bradshaw, pers. comm.).

**Figure 7:**
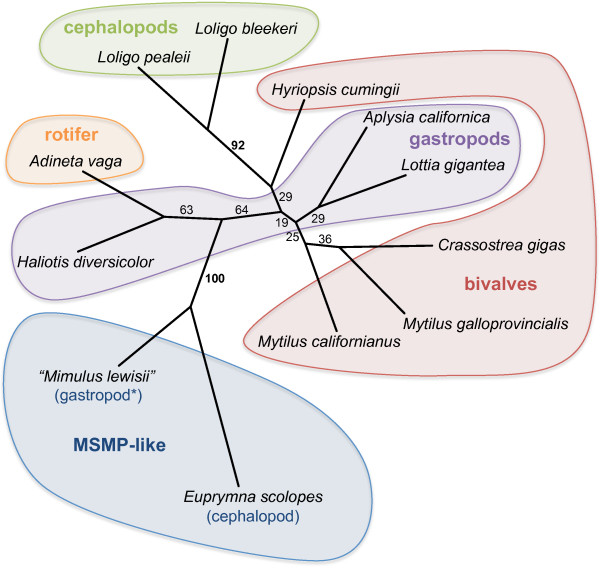
**Phylogenetic analysis of *MSMB*-related genes in protostomes**. Translated sequences spanning only from the N-terminus of the secreted protein to the last conserved cysteine residue were aligned. The unrooted tree was produced by the neighbor-joining method with 1000 replicates. Significant bootstrap values (> 70%) are shown in bold. * The sequence for *"Mimulus lewisii*" is presumed to represent a terrestrial gastropod (see text).

Phylogenetic analysis of all the protostomal *MSMB*-like translated sequences did not entirely reflect the species' taxonomic relationship (Figure 7), suggesting that not all the sequences are orthologous to each other. Most notably, the similarity between a sequence from a cephalopod, *Euprymna scolopes*, and the "*Mimulus lewisii*" (presumed terrestrial gastropod) sequence is stronger (100% bootstrap) than between any other sequence pairs, including between congeneric species (92% for *Loligo spp*.; 25% for *Mytilus spp*.). This suggests that the former sequences represent a gene that is subject to more evolutionary constraints, similarly to *MSMP *in vertebrates. Indeed, the sequences from *Euprymna scolopes *and "*Mimulus lewisii*" appear to share some features that are highly conserved in vertebrate MSMPs, such as a serine-alanine motif near the C-terminus (not shown). Thus it is possible that these two sequences represent distant orthologues of *MSMP*.

The California sea hare (*Aplysia californica*; Gastropoda) is the only mollusc currently with a WGS sequencing project. The sea hare *MSMB*-like sequence is located on the same genomic scaffold (Scaffold 217 of genome build *Broad 2.0/aplCal1*) as a member of the *KLHL *(Kelch-like) gene family. *KLHL *genes also respectively occupy the syntenic groups that include *MSMB *or *USM/USMH *in vertebrates (not shown). Together these data suggest a divergence between the *MSMP *and *USM/MSMB *lineages in a bilaterian common ancestor.

### Predicted tertiary structure of marsupial USM

**Figure 8:**
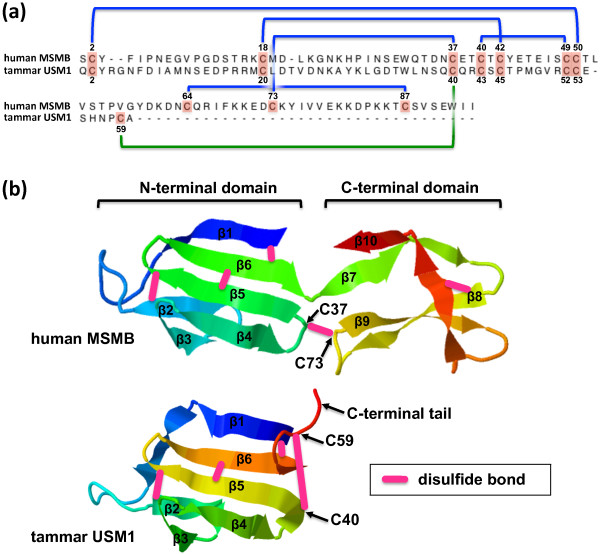
**Predicted tertiary structure of tammar USM1 modelled on human MSMB**. (a) The secreted forms of human MSMB and tammar USM1 (predicted) are aligned with the N-terminal domain (above) and the C-terminal domain (below). The ten cysteine residues in MSMB (highlighted in pink with positions indicated) form five disulfide bonds as shown (blue lines). Cysteine residues homologous to C64, C73 and C87 are absent in USM1 due to its C-terminal truncation, leaving C40 in USM1 (homologous to C37 in MSMB) unpaired. We suggest that an additional C-terminal cysteine residue (C59), also conserved in brushtail possum and opossum, instead forms a disulfide bond with C40. (b) The secreted forms of human MSMB (monomer) and tammar USM1 modelled on the crystal structure of human MSMB [35] (Protein Data Bank accession code 3IX0) using ESyPred3D [65,66] and displayed as a cartoon with β strands in rainbow colour using Jmol [67]. The approximate positions of disulfide bonds are indicated by pink bars. Predicted disulfide bonds in tammar USM are shown in homologous positions. It is evident that C40 and C59, which lies within the short, flexible C-terminal tail, are in close proximity and likely to form a disulfide bond.

Almost all USM/MSMB/MSMP family members share a conserved pattern of ten disulfide-forming cysteine residues, whereas marsupial USM has only eight cysteine residues due to a truncated reading frame in Exon 4 (Figure 8a). The disulfide bond pairings of cysteine residues has been partially determined [15] and then later refined [33,34]. A recent crystallographic analysis of human MSMB [35] showed that the N-terminal domain consists of six β-strands (β1-6) arranged in a Greek key motif, while the C-terminal domain consists of four β-strands (β7-10). Three disulfide bonds (6 cysteine residues) give rigidity within the N-terminal domain and one disulfide bond (2 cysteine residues) gives rigidity within the C-terminal domain. A fifth, single disulfide bond between C37 and C73 links the N-terminal and C-terminal domains. In marsupial USM, the cysteine residue homologous to C37 (= C40 in tammar secreted USM1) is conserved, despite the absence of C73. However, an additional cysteine residue (= C59 in the tammar secreted protein) is present within the short, six-residue C-terminal domain of all marsupial USM orthologues. Modelling of the tammar USM1 tertiary structure showed that this cysteine residue would lie very close to C37 and substitute for the missing C73 (Figure 8b).

The crystal structure of MSMB also revealed a mechanism for dimerisation, whereby the β1 and β10 strands of one molecule lie end-to-end to form a straight edge which lies antiparallel and in contact with the β1 and β10 strands of a second molecule [35]. The involvement of both β1 (N-terminal domain) and β10 (C-terminal domain) strands suggests that dimerisation cannot occur in USM, which lacks sequence homologous to β10. This might be integral to a divergent role for USM compared with MSMB and USMH. However, the molecular masses of bands 3-5 in the original protein gel of [2] (Figure 1), which each contained USM sequence, were estimated by the authors as 22, 17 and 14 kDa, respectively. Bands 3 and 5 are thus approximately three- and two-fold, respectively, the predicted molecular mass of monomeric secreted USM (7 kDa). It thus remains possible that USM can form multimers despite its C-terminal truncation. It is noteworthy that the immunoglobulin-binding property of MSMB may depend on dissociation of dimers to monomers in response to reducing conditions or low pH [35,36]

The precise role of USM in the marsupial postovulatory coats is an intriguing question considering the various roles that have been proposed for MSMB. While MSMB was first identified almost three decades ago as a component of human seminal plasma with FSH-inhibiting activity [16], there has been a recent resurgence in interest due to a demonstrated genetic link with prostate cancer susceptibility [37-40]. Perhaps more relevant to the present context, MSMB has been shown to inhibit sperm binding and the acrosome reaction [11,41], suggestive of a possible role in blocking polyspermy in marsupials.

### Expression of USM, MSMB and MSMP in tammar tissues

**Figure 9:**
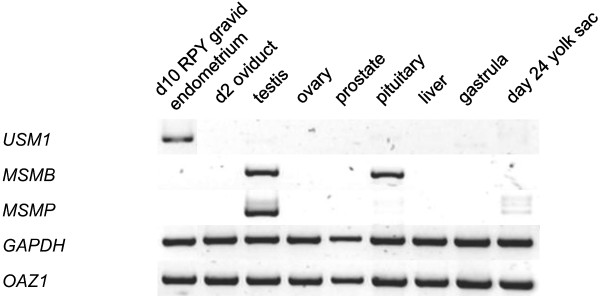
**RT-PCR expression of tammar *USM1, MSMB1 *and *MSMP *in various tissues**. *USM1 *expression was detected more strongly in endometrium but not in any other tissues, consistent with a specific role in the postovulatory coats. *MSMB1 *and *MSMP *were both expressed in testis, while *MSMB1 *was also expressed in the pituitary gland, consistent with a possible role in the pituitary-gonadal axis.

To elucidate distinctions in the roles of *USM, MSMB *and *MSMP *in the tammar, RT-PCR was performed on a variety of tissues (Figure 9). *USM1 *expression was detected solely within endometrium and not in other tissues, including oviduct. This is consistent with a highly specific role for USM1 as a component of the postovulatory coats. *MSMB *expression was detected in both pituitary gland and testis, while *MSMP *expression was restricted to testis only. Expression of *USM2 *was not detected in the tissues tested.

**Figure 10:**
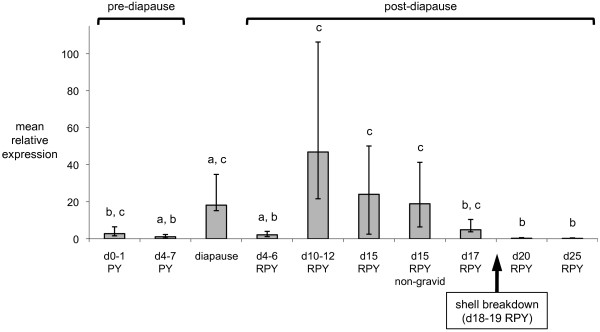
**Quantitative RT-PCR analysis of *USM1 *expression in endometrium during tammar development**. Pre-diapause stages are indicated by the age of the pouch young (PY) arising from the previous pregnancy, with blastocysts entering diapause when PY are around day 8 (d8). Post-diapause stages are indicated by the days after removal of pouch young (RPY) to initiate blastocyst reactivation. *USM1 *shows dynamic changes in expression during the pregnant oestrous cycle. The large reduction in expression to basal levels after d17 RPY correlates approximately with the period of shell breakdown (d18-19 RPY) as indicated.

Expression of *USM1 *was examined by quantitative RT-PCR during gestation (Figure 10). Transcript levels were moderate-to-high during pre-diapause (days 0-7 after birth of previous young), diapause, and early post-diapause (until day 17-18 after blastocyst reactivation by removal of pouch young (RPY)). Levels were higher during diapause than pre-diapause and the earliest post-diapause stages, although these differences were not significant. By contrast, the second peak at around d10-15 RPY was significantly higher than d4-d6 RPY and d20-25 RPY. After d15 RPY there was a rapid reduction in USM1 transcript levels, which coincides with shell breakdown at around d18-19 RPY [7].

The dynamic temporal expression pattern of *USM1 *during gestation suggests that it may be at least partly regulated by progesterone - indeed we identified a progesterone receptor binding site within the first intron that was conserved in both tammar and opossum (not shown). In the tammar, progesterone receptors are highest at around d5 RPY, together with oestrogen receptors, coinciding with the progesterone and oestrogen pulses that occur at this time [42]. Interestingly this is exactly when *USM1 *is at its lowest level before increasing again. *USM1 *expression is also low after d20 of pregnancy (Figure 10), at the time when progesterone concentrations in the corpus luteum [43], in the peripheral circulation [44], and in the utero-ovarian circulation [45] are highest, but progesterone receptor levels are very low [42]. Thus, *USM1 *expression appears to follow the profile of progesterone receptor levels rather than of progesterone.

### USM as a component of the marsupial postovulatory coats

The mucoid layer is deposited during passage through the oviduct whereas shell coat material is secreted from endometrial glands within the uterus and the uterotubal junction. The association of MSMB expression with mucosal epithelia [46] suggests that USM might contribute to the mucin layer, however, the brushtail possum protein bands isolated by Casey *et al*. [2] were derived from a mixed pool of coats from early cleavage through to late expansion conceptuses. Coats of the former would be expected to include more mucoid coat while the latter would include more shell coat. The much greater volume of shell coat surrounding late-expansion conceptuses [5] suggests that shell coat material would predominate in any mixed pool of samples. Indeed, expression in the endometrium but not the oviduct revealed by our RT-PCR data is consistent with contribution to the shell coat rather than the mucin layer, which forms first in the oviduct. Nevertheless, there may be some overlap in the components of both layers, with the physical differences between the shell and mucin coat due to a subset of components that are specific to one or the other. Immunolocalisation studies may clarify the relative contribution of USM to each layer.

Many of the known properties of MSMB provide clues as to possible role(s) of USM. In marsupials, intimate contact between conceptus and maternal tissues occurs only late in development, after shell breakdown approximately two-thirds of the way through pregnancy [47,48]. The binding of MSMB to immunoglobulins [12] suggests that USM, if it shares this property, may interact with the maternal immune system to modulate its action. The apparently synchronised down-regulation of tammar *USM1 *with shell breakdown suggests that the former may be a necessary step for subsequent successful implantation. Alternatively, *USM1 *down-regulation might facilitate shell-breakdown itself.

USM could also have an immune role in protection against pathogens within the uterus. We have identified a lysozyme as another component of the postovulatory coats (unpublished data) that may have a similar role in protection against bacteria. Such a role for USM would be consistent with the association of MSMB secretion in mucosal tissues [49]. In eutherians, degradation of mucin on the endometrial surface is associated with a window of receptivity to implantation (reviewed [50]). Thus it is possible that similar events, including down-regulation of USM expression, are associated with placental attachment after shell breakdown in marsupials.

Unlike MSMP, which is relatively well conserved, MSMB is characterised by a rapid rate of evolution. Mäkinen *et al*. [27] noted that among the multiple copies of *MSMB *in New World monkeys, the second intron is more highly conserved than the exons and there is no bias towards substitutions in the third nucleotide of codons, which normally preserve amino acid identity. Thus it is possible that rapid change in the primary structure of MSMB, excluding the signal peptide and the cysteine residues, is under positive selection. In another study [51], *MSMB *was identified in a screen for prostate-expressed genes in primates with a high ratio of nonsynonymous to synonymous substitution rate (*d*_N_/*d*_S_) - a conservative measure of positive selection. USM is similarly highly divergent among the three marsupial species examined and, like MSMB, its divergence might be due to positive rather than neutral selection. It was previously proposed that MSMB prevents immune attack against allogeneic sperm [12]. A possible extension to this idea may be that the same mechanism also serves to reject heterospecific sperm, as MSMB has been shown to bind sperm and act as an inhibitor of sperm motility and the acrosome reaction [10,11,41]. Thus rapid evolution of USM (and MSMB) could be implicated in speciation events by preventing hybridisation with closely related species. A recent report [24] showed that male longfin inshore squid (*Loligo pealeii*) detect an MSMB-like protein, *Loligo *β-MSP, in the capsule of eggs laid on the sea floor. *Loligo *β-MSP triggers hostile behaviour in conspecific males towards other male squid, demonstrating a possible role in species recognition. It is not clear whether this reflects only a secondary role for *Loligo *β-MSP in the egg capsule, but the parallel with USM as a component of the marsupial conceptus coats is intriguing, although in marsupials internal fertilisation and development rules out any role for USM in mate selection by males. However, it remains possible that USM within the reproductive tract helps to ensure fertilisation by only con-specific sperm.

Some eutherians such as rabbit, horse and some carnivores (reviewed [52-54]) also possess various post-ovulatory conceptus coats, such as a mucoid coat, neozona and gloiolemma (rabbit) or a capsule (horse). It is not known whether any components of marsupial and eutherian mucoid coats are homologous, but our thorough searches in both rabbit and horse genome databases suggest that no orthologues of *USM *are present. However, other components could be homologous. It also cannot be excluded that MSMB or MSMP have acquired an analogous role in the coats of some eutherians by convergent evolution. It is noteworthy that *MSMB *expression has also been detected in human endometrium [55].

Very few genes have been identified in marsupials that are absent in eutherian genomes [9]. The identification of *USM *is thus noteworthy and could be highly relevant to understanding the differences in modes of reproduction between these two major mammalian groups. If USM homologues in non-mammalian vertebrates have a different role to marsupial USM, this would suggest that the latter evolved in concert with mammalian viviparity by supporting *in utero *development. Conversely, the absence of USM in eutherians suggests the evolution of alternate mechanisms supporting *in utero *development that caused USM to be redundant, for presumably the same reason that the shell coat became redundant.

## Conclusions

Very few genes have been identified that are specific to marsupials, one of the three major extant groups of mammals. We have identified one such gene - *USM *- and attributed to its product a role in the postovulatory coats of the marsupial conceptus. Its likely importance in reproduction has potential applications in fertility control and its high sequence divergence may facilitate species-specificity when targeting wild populations. We have also provided the most comprehensive analysis to date of the complex evolutionary relationships between different members of the vertebrate *USM-MSMB-MSMP *gene family. Despite more than 30 years since the initial identification of MSMB, this gene family remains poorly understood. It has attracted attention in multiple, diverse fields of research, including immunity, reproduction, sexual selection and cancer. Our results provide valuable information that may help to elucidate not only the evolution of viviparity and placental function in mammals, but also the roles of MSMB and MSMP.

## Methods

### Animals and tissue sampling

Tammar samples were collected from animals shot on Kangaroo Island, South Australia, or from captive animals from a colony maintained by the University of Melbourne. Tissues from adult tammars were frozen in liquid nitrogen. All tissues were collected under appropriate permits. Experiments were approved by the University of Melbourne Animal Experimentation Ethics Committee and all animal handling and husbandry was in accordance with the National Health and Medical Research Council of Australia (2004) guidelines.

### RNA extraction and reverse transcription

Total RNA was extracted using Tri Reagent (Ambion) and DNase-treated using DNA-*free *(Ambion). Reverse transcription was performed using the Transcriptor High Fidelity cDNA Synthesis Kit (Roche) with oligo-dT priming.

### RT-PCR

The complete coding region of tammar *USM1 *cDNA was amplified using the primers 5'-GGGGCACGAATGGGTGTTTATTC-3' and 5'-CCTGAGACACAGAGGAACCAGAGGTACTG-3' and TaKaRa *ExTaq *polymerase according to the manufacturer's protocol. The PCR product was cloned using the pGEM-T-Easy kit (Promega) and sequenced using vector-specific primers. The transcription start site was identified by nested 5' RACE using the SMARTer RACE cDNA Amplication Kit (ClonTech) and the reverse primers 5'-CCTGAGACACAGAGGAACCAGAGGTACTG-3' (first PCR) and 5'-CAGACCAATCAGACGCTCC-3' (nested PCR). The purified 5' RACE product was directly sequenced using the nested reverse primer.

Semi-quantitative RT-PCR on cDNA from adult tissues was performed using the following primers. *USM1 *- 5'-GGGGCACGAATGGGTGTTTATTC and CCTGAGACACAGAGGAACCAGAGGTACTG; *USM2 *- 5'-TGTTGGCAAGAAGGGTCAATGTCC-3' and TTCCTGAGAGGTACAGGTGTCAGTTATGC-3'; *MSMB1 *- 5'-GATTGCTGCTGGTCTCGTGACTACTG-3' and AGGATTTGGTGGGGTCTTCTTTATGC; *MSMP *- 5'-GGTAGTGGTCAATGGAGTTGCTGATGC-3' and 5'-ACTTCGGAGCCAGGATTCACCC-3'; *GAPDH *- 5'-CCTACTCCCAATGTATCTGTTGTGG-3' and 5'-GGTGGAACTCCTTTTTTGACTGG-3'. Amplification was performed using the following programme: 95°C for 1 minute; 35 cycles of 95°C for 15 seconds, 60°C for 15 seconds, 72°C for 30 seconds; 72°C for two minutes.

### Quantitative RT-PCR

Quantitative RT-PCR (qRT-PCR) was performed using the Brilliant II SYBR Green qPCR Kit (Agilent Technologies) and reactions run in triplicate on an Mx3000P thermal cycler (Stratagene). The quantities of tammar *USM1 *transcripts, using the primers 5'-GGGGCACGAATGGGTGTTTATTC-3' and 5'-CCTGAGACACAGAGGAACCAGAGGTACTG-3', were compared to the housekeeping gene *β-ACTIN*, using the primers 5'-TTGCTGACAGGATGCAGAAG-3' and 5'-AAAGCCATG-CCAATCTCATC-3'. Amplification was performed using the following program: 95°C for 15 minutes; 50 cycles of 95°C for 15 seconds, 60°C for 30 seconds (which included the plate read) and 72°C for 30 seconds. This was followed by 95°C for 1 minute and a dissociation curve of 55°C to 95°C, with a reading every 0.5°C over 30 seconds.

Plates were discarded if more than one of the negative control triplicates was contaminated. Individual samples of a triplicate were also discarded if they had irregular melting curves or if the coefficient of variation was greater than 0.05 for the triplicate. If more than one of the triplicates was irregular the sample was repeated or discarded. In addition, a calibrator sample (d4-5 PY stage gravid endometrium) was also run across all plates to control for inter-assay variation, and the efficiency of each primer set was also determined. Analysis of the data was based on a modification of the 'efficiency-corrected comparative quantification method', which incorporates both the individual efficiencies of each primer set and the calibrator sample into the calculations [56]. This gave a normalised relative quantity value for each sample, which was then used for the subsequent analysis.

Statistical analyses were conducted using R (version 2.11.1) [57]. A Shapiro-Wilks test for normality was performed to check the assumption that the data had a normal distribution. Since the distribution of the relative expression values was skewed, the data were log transformed for analysis. Log transformed data was analysed by one-way ANOVA with multiple comparisons of means compared using Tukey contrasts. For all analyses a significance level of p < 0.05 was used. Data are presented as mean ± SEM after converting back to non-transformed normalised relative expression.

### Bioinformatics

Sequence searches of Whole Genome Shotgun, Expressed Sequence Tag and Nucleotide databases were performed through the National Center for Biotechnology Information (NCBI) website [58] using BLAST and tBLASTn and modifying search parameters for stringency. Nucleotide and translated sequences were analysed using the MacVector sequence analysis software package. Protein molecular masses were predicted using MacVector's Protein Analysis Toolbox. Signal peptide cleavage sites were identified using the SignalP 3.0 web-based software [59] with default parameters.

### BAC identification and sequencing

A tammar BAC genomic library (MEB1) was screened by PCR using primers 5'- GGGGCACGAATGGGTGTTTATTC -3' and 5'- GGAAGAGTGGAGGATGGATTTGAGG -3'. Illumina 454 sequencing was performed by the Australian Genome Research Facility. The longest assembled contig (89, 803 nucleotides) was submitted to GenBank [accession JN251945].

### Phylogenetic analysis

Translated sequences were aligned using ClustalW [60] within the MacVector sequence analysis software package. The unrooted phylogenetic tree was produced using Phylip (version 3.69) software [61] and running sequentially the programs protdist, neighbor and drawtree with default parameters. The tree was displayed and edited using Adobe Illustrator.

### Promoter analysis

Promoter analysis was performed by aligning tammar *USM1 *and opossum *USM *genomic sequences using Mulan online software http://mulan.dcode.org/[62,63] in 'TBA' mode. Conserved candidate transcription factor binding sites were identified using multiTF [63,64] from the Mulan website, selecting the TRANSFAC professional V10.2 TFBS database for vertebrates, and selecting the "optimised for function" option for matrix similarity.

## Authors' contributions

SF performed bioinformatical analyses, semiquantitative RT-PCR and cloning. JF performed quantitative RT-PCR. BD performed BAC library screening. SF, JF, GS and MBR analysed data and prepared the manuscript. All authors read and approved the final manuscript.

## Supplementary Material

Additional file 1 **Alignment of translated sequences of selected members of the *USM/MSMB/MSMP *family**. Protein sequences are grouped according to sub-family. Boundaries between regions encoded by Exons 2, 3 and 4 are indicated by orange lines. Shading indicates identity in at least 50% of sequences. The predicted signal peptide cleavage site for USM orthologues is indicated by an arrowhead. Click here for file

Additional file 2 **Sources of sequences used in this study**. Transcript sequence sources are provided either as GenBank or UniGene accession numbers. Coding regions predicted from this study are defined by nucleotide ranges within genomic sequences, either from genome builds or whole genome shotgun scaffolds or contigs, as specified. Exons 1-4 refer for MSMP are defined for ease of comparison by their homology with Exons 1-4 of human *MSMB*. Click here for file

Additional file 3 **Phylogenetic tree of microproteins from chordates using an alignment of protein sequences**. The unrooted tree was constructed by aligning translated sequences homologous to Exons 3-4 of mouse *Msmb *and subjecting the alignment to the program Protpars (Phylip) followed by Drawtree. Most branch points do not yield significant bootstrap values using various methods (not shown), but sequences generally cluster into the three groups highlighted. Accordingly, the position of zebrafish usmh1 in the tree is unlikely to reflect its true phylogeny, as predicted by its conserved synteny. Click here for file

Additional file 4 **Amino acid identity and similarity matrices for avian MSMB1, MSMB2 and MSMB3 and mouse Msmb**. Highlighted cells refer to values cited in the text. Yellow: percentage identities between each zebra finch paralogue and its orthologues in other species are highest for MSMB3. Blue: percentage identities between the turkey and chicken orthologues of each paralogue are also highest for MSMB3. Orange: mouse Msmb shows higher identity to MSMB2 than to either MSMB1 or MSMB3. Click here for file
