# Supplementary material for: A novel MSMB-related microprotein in the postovulatory egg coats of marsupials
Source: BMC Evol Biol. 2011 Dec 30;11:373. doi: 10.1186/1471-2148-11-373 (PMC3268785; doi:10.1186/1471-2148-11-373)
Supplement: Additional file 1 — Alignment of translated sequences of selected members of the USM/MSMB/MSMP family. Protein sequences are grouped according to sub-family. Boundaries between regions encoded by Exons 2, 3 and 4 are indicated by orange lines. Shading indicates identity in at least 50% of sequences. The predicted signal peptide cleavage site for USM orthologues is indicated by an arrowhead. [file 1471-2148-11-373-S1.PDF]

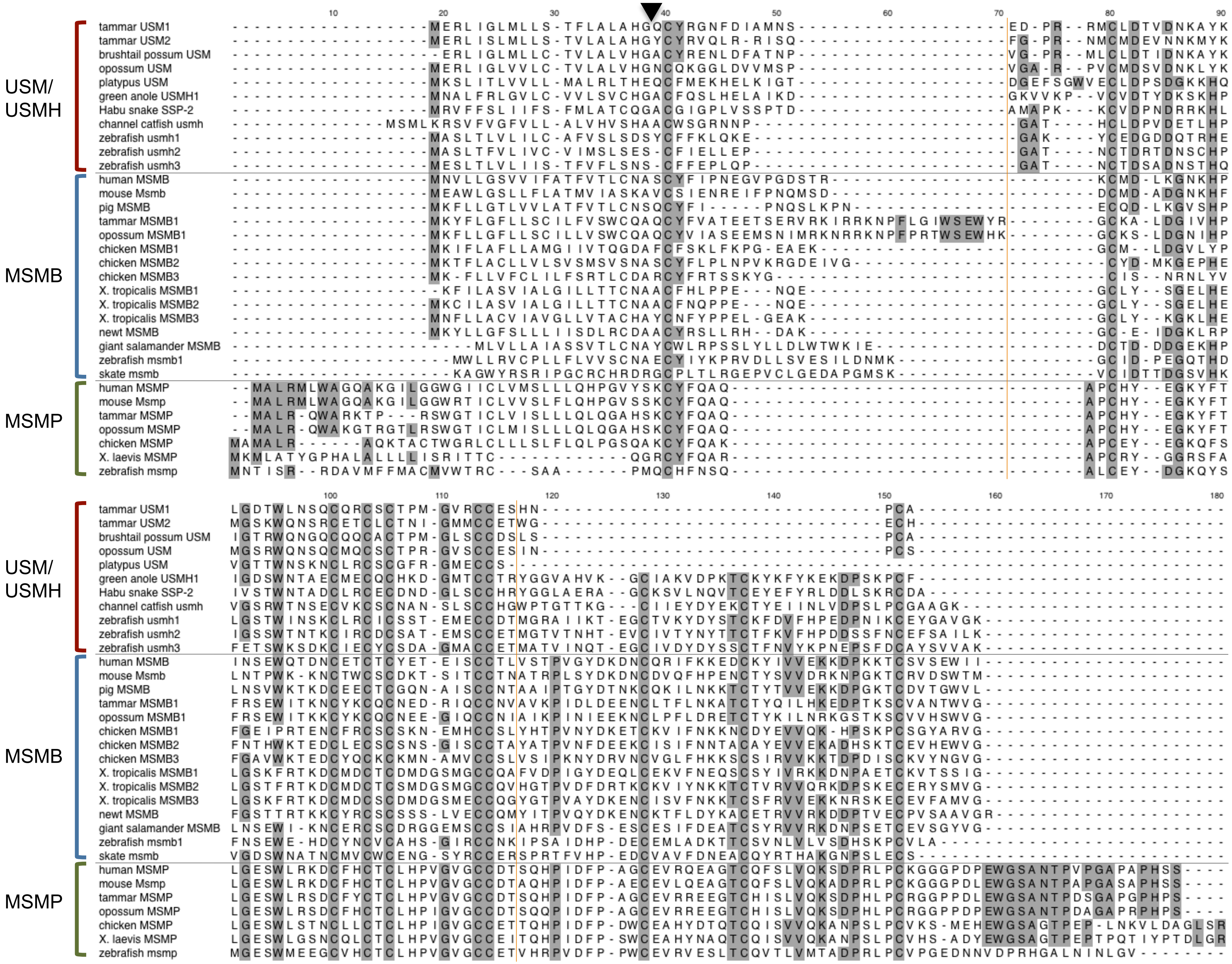
USM/
USMH
MSMB
MSMP
tammar USM1
tammar USM2
brushtail possum USM
opossum USM
platypus USM
green anole USMH1
Habu snake SSP-2
channel catfish usmh
zebrafish usmh1
zebrafish usmh2
zebrafish usmh3
human MSMB
mouse Msmb
pig MSMB
tammar MSMB1
opossum MSMB1
chicken MSMB1
chicken MSMB2
chicken MSMB3
X. tropicalis MSMB1
X. tropicalis MSMB2
X. tropicalis MSMB3
newt MSMB
giant salamander MSMB
zebrafish msmb1
skate msmb
human MSMP
mouse Msmp
tammar MSMP
opossum MSMP
chicken MSMP
X. laevis MSMP
zebrafish msmp
10
20
30
40
50
60
70
80
90
100
110
120
130
140
150
160
170
180
