# Supplementary material for: A novel MSMB-related microprotein in the postovulatory egg coats of marsupials
Source: BMC Evol Biol. 2011 Dec 30;11:373. doi: 10.1186/1471-2148-11-373 (PMC3268785; doi:10.1186/1471-2148-11-373)
Supplement: Additional file 2 — Sources of sequences used in this study. Transcript sequence sources are provided either as GenBank or UniGene accession numbers. Coding regions predicted from this study are defined by nucleotide ranges within genomic sequences, either from genome builds or whole genome shotgun scaffolds or contigs, as specified. Exons 1-4 refer for MSMP are defined for ease of comparison by their homology with Exons 1-4 of human MSMB. [file 1471-2148-11-373-S2.PDF]

Additional file 1: sequence sources

species	gene name	UniGene accession	GenBank accession	genome build	Chromosome/ Scaffold/Contig	predicted coding region from genomic sequences			
						Exon 1	Exon 2	Exon 3	Exon 4
Crassotrea gigas **(Pacific oyster)**			CU986619						
Hyriopsis cumingii **(triangle shell mussel)**			GW693606						
Mytilus californianus **(California mussel)**			GE747557						
Mytilus galloprovincialis **(Mediterranean mussel)**			AJ625850						
Aplysia californica **(Californian sea hare)**			GD196606						
Haliotis diversicolor **(abalone)**			GT870297						
Lottia gigantea **(owl limpet)**			FC696420						
"Mimulus lewisii" **(probable terrestrial gastropod)**			GR204924						
Adineta vaga **(rotifer)**			ACF16060						
Euprymna scolopes **(Hawaiian bobtail squid)**			DW282367						
Loligo bleekeri **(Bleeker's squid)**			FS372646						
Loligo pealeii **(longfin inshore squid)**			GQ906708						
Ciona intestinalis **(sea squirt)**	*msmb-like*				AABS01000004			243712-243856	244207-244465
	msmp		BP016804						
Branchiostoma belcheri tsingtauense **(lancelet)**			AY551566						
Petromyzon marinus **(lamprey)**	*msmp*		DY250259						
Leucoraja erinacea **(little skate)**			GD274048						
Danio rerio **(zebrafish)**	*msmb1*	Dr.86797		Zv9/danRer7	Chr. 17				
	msmb2	Dr.91643		Zv9/danRer7	Chr. 17				
	msmb3	Dr.154450		Zv9/danRer7	Chr. 17				
	umph1	Dr.77131		Zv9/danRer7	Chr. 8				
	umph2	Dr.113263		Zv9/danRer7	Chr. 8				
	umph3	Dr.116246		Zv9/danRer7	Chr. 8				
	msmp	Dr.115744		Zv9/danRer7	Chr. 7				
Ictalurus punctatus **(channel catfish)**			GH690284						
Notophthalmus viridescens **(newt)**	*MSMB*		GO933280						
Andrias davidianus **(chinese salamander)**	*MSMB*		EH112545						
Rana chensinensis **(Chinese brown frog)**	*MSMB*		FD447660						
Xenopus tropicalis **(Western clawed frog)**	*MSMB1*	Str.74314	XM_002935969	JGI 4.1/xenTro2	Scaffold 179				
	MSMB2	Str.32348	XM_002935961	JGI 4.1/xenTro2	Scaffold 179				
	MSMB3	Str.41109	XM_002935960	JGI 4.1/xenTro2	Scaffold 179				
	MSMB4	Str.41789	XM_002935959	JGI 4.1/xenTro2	Scaffold 179				
Xenopus laevis **(African clawed frog)**	*MSMP*	Xl.59761	NM_001135233		Scaffold 1451				
Trimeresurus flavoviridis **(habu snake)**	*SSP-1*		AB360906						
	SSP-2		AB360907						
	SSP-3		AB360908						
	SSP-4		AB360909						
	SSP-5		AB360910						
Anolis carolinensis **(green anole)**	*USMH1*			Broad/anoCar1	Scaffold 572	686571-686573	687957-688050	689695-689821	691094-691199
	USMH2		FG730785	Broad/anoCar1	Scaffold 572	698638-698640	704724-704817	706975-707101	708744-708849
	USMH3		FG759575	Broad/anoCar1	Scaffold 572				728862-728970
	USMH4			Broad/anoCar1	Scaffold 572			735396-735525	
	USMH5			Broad/anoCar1	Scaffold 572			739481-739352	737124-737010
	USMH6			Broad/anoCar1	Scaffold 572			748552-748681	
	USMH7		FG742191	Broad/anoCar1	Scaffold 572	752468-752470	754316-754409	757215-757344	
Anas platyrhynchos **(mallard duck)**	*MSMB3*		HO188240						
Gallus gallus **(chicken)**	*MSMB1*	Gga.29680		WUGSC 2.1/galGal3	Chr. 6				
	MSMB2	Gga.5954		WUGSC 2.1/galGal3	Chr. 6				
	MSMB3			WUGSC 2.1/galGal3	Chr. 6	18925393-18925395	18925567-18925651	18926352-18926454	18926905-18927034
	MSMP		XM_001233140	WUGSC 2.1/galGal3	Chr. 6				
Meleagris gallopavo **(turkey)**	*MSMB1*					ADDD01063179: 2080-2078	ADDD01063179: 533-425	ADDD01009838: 14249-14140	ADDD01009838:13448-13319
	MSMB2		ADDD01126309			5464-5462	3165-3066	2540-2438	1576-1440
	MSMB3					ADDD01009838: 5731-5729	ADDD01009838: 5552-5468	ADDD01009838: 4781-4679	ADDD01009838: 4227-4098
Taeniopygia guttata **(zebra finch)**	*MSMB1*			WUGSC 3.2.4/taeGut1	Chr. 6	16862426-16862428	16863454-16863562	16864800-16864905	16865589-16865718
	MSMB2			WUGSC 3.2.4/taeGut1	WGS Contig16004.1	4381-4379	2095-1996	1676-1574	713-584
	MSMB3			WUGSC 3.2.4/taeGut1	Chr. 6	16872846-16872848	16873007-16873097	16873777-16873879	16875521-16875650
Struthio camelus **(ostrich)**	*MSMB2*		P83242 (protein)						
Ornithorhynchus anatinus **(platypus)**	*USM*					AAPN01348068: 5128-5059	AAPN01348068: 2075-1982	WGS trace 686563447: 479-611	
Trichosurus vulpecula **(brushtail possum)**	*USM*		EG617409						
Macropus eugenii **(tammar wallaby)**	*USM1*		JN251945			12801-12889	17277-17370	18318-18441	19044-19128
	USM2		JN251945			28884-28970	40280-40373	43621-43741	44697-44715
	MSMB1						ABQO011055171: 614-767	ABQO010035330: 519-624	WGS trace 1283620425: 723-593
	MSMB2		ABQO010216845		WGS Contig216854			2387-2392	3490-3541
	MSMB3		ABQO010400686		WGS Contig400704			1531-1636	2784-2913
	MSMB4		ABQO010518578		WGS Contig518601			996-1101	2225-2354
	MSMB5		ABQO010335897		WGS Contig335912			820-925	2058-2277
	MSMB6		ABQO010234570		WGS Contig234579			749-647	
	MSMB7		ABQO010907673		WGS Contig907710			342-447	
	MSMB8				WGS trace 1603589103			688-793	
	MSMB9		ABQO010137468		WGS Contig137472			670-565	
	MSMB10		ABQO010137466		WGS Contig137470			1623-1728	
	MSMP						ABQO010874819: 672-555	ABQO010874819: 295-187	ABQO010773949: 2016-2236
Monodelphis domestica **(grey short-tailed opossum)**	*USM*			Broad/monDom5	Chr. 1	589655115-589655027	589648051-589647958	589646741-589646618	589646259-589646237
	MSMB1			Broad/monDom5	Chr. 1	13790901-13790899	13785351-13785198	13775863-13775758	13770408-13770279
	MSMB2			Broad/monDom5	Chr. 1		13833835-13833730	13832924-13832816	13829089-13828960
	MSMB3			Broad/monDom5	Chr. 1			14001460-14001355	14000012-13999886
	MSMB4			Broad/monDom5	Chr. 1			14044556-14044451	14043153-14043024
	MSMB5			Broad/monDom5	Chr. 1			14072372-14072267	14070960-14070831
	MSMB6			Broad/monDom5	Chr. 1			14090464-14090359	14089073-14088944
	MSMB7			Broad/monDom5	Chr. 1			14110575-14110470	14109180-14109054
	MSMB8			Broad/monDom5	Chr. 1			14141928-14141823	14140516-14140390
	MSMB9			Broad/monDom5	Chr. 1			14209921-14209816	14208511-14208382
	MSMB10			Broad/monDom5	Chr. 1			14237311-14237206	14235909-14235780
	MSMB11			Broad/monDom5	Chr. 1			14248458-14248353	14247024-14246934
	MSMB12			Broad/monDom5	Chr. 1			14276415-14276310	14274892-14274763
	MSMB13			Broad/monDom5	Chr. 1			14319112-14319007	14317581-14317452
	MSMB14			Broad/monDom5	Chr. 1				14335017-14334888
	MSMP				Chr. 6		122604600-122604726	122605008-122605116	122605413-122605593
Mus musculus **(mouse)**	*MSMB*		NM_020597						
	MSMP		NM_001099314						
Sus scrofa **(pig)**	*MSMB*		NM_213852						
Homo sapiens **(human)**	*MSMB*		NM_002443						
	MSMP		NM_001044264						
