# Supplementary figures and images for: A novel MSMB-related microprotein in the postovulatory egg coats of marsupials
Source: BMC Evol Biol. 2011 Dec 30;11:373. doi: 10.1186/1471-2148-11-373 (PMC3268785; doi:10.1186/1471-2148-11-373)

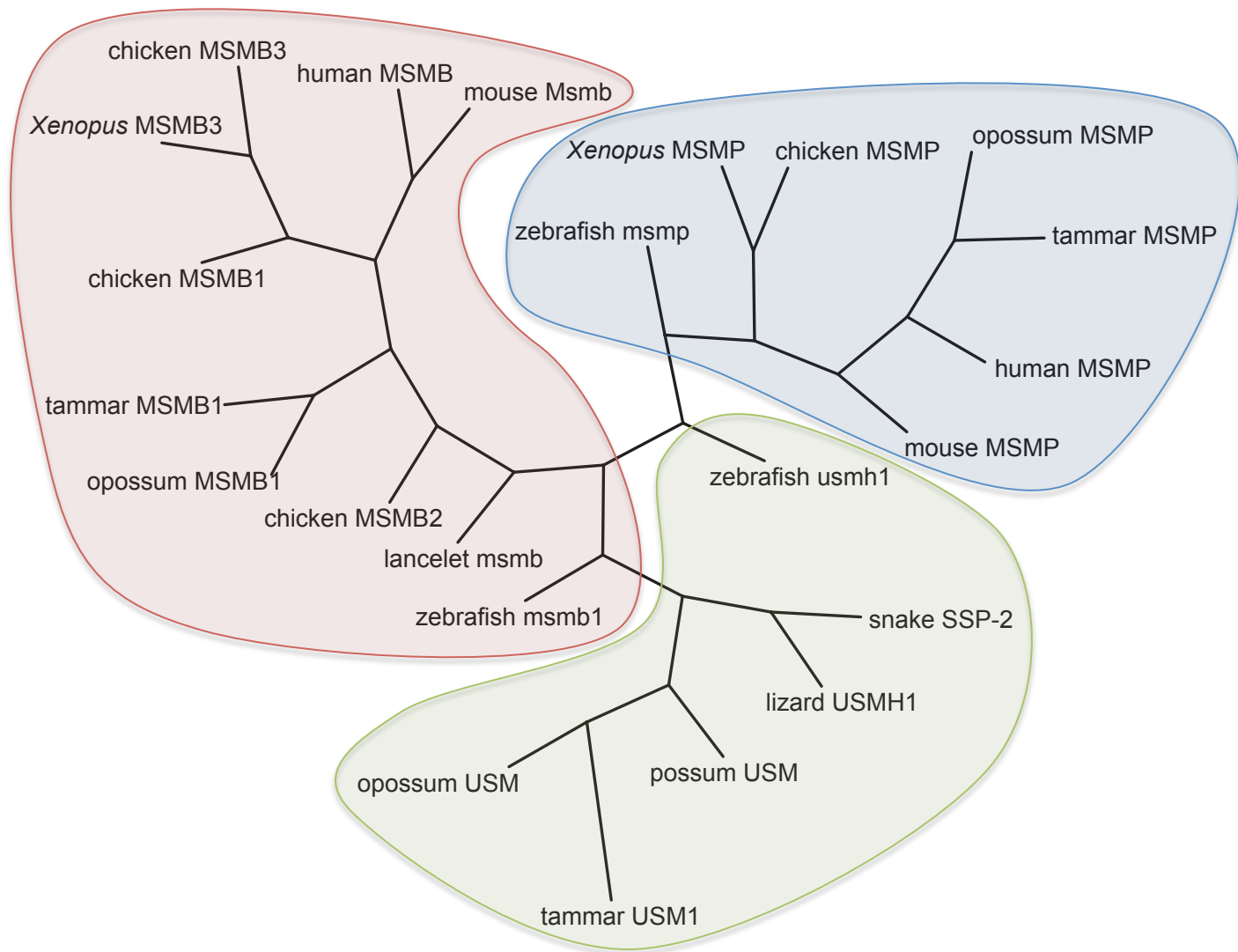

Supplement: Additional file 3 — Phylogenetic tree of microproteins from chordates using an alignment of protein sequences. The unrooted tree was constructed by aligning translated sequences homologous to Exons 3-4 of mouse Msmb and subjecting the alignment to the program Protpars (Phylip) followed by Drawtree. Most branch points do not yield significant bootstrap values using various methods (not shown), but sequences generally cluster into the three groups highlighted. Accordingly, the position of zebrafish usmh1 in the tree is unlikely to reflect its true phylogeny, as predicted by its conserved synteny. [file 1471-2148-11-373-S3.PDF]
