# Supplementary material for: A novel MSMB-related microprotein in the postovulatory egg coats of marsupials
Source: BMC Evol Biol. 2011 Dec 30;11:373. doi: 10.1186/1471-2148-11-373 (PMC3268785; doi:10.1186/1471-2148-11-373)
Supplement: Additional file 4 — Amino acid identity and similarity matrices for avian MSMB1, MSMB2 and MSMB3 and mouse Msmb. Highlighted cells refer to values cited in the text. Yellow: percentage identities between each zebra finch paralogue and its orthologues in other species are highest for MSMB3. Blue: percentage identities between the turkey and chicken orthologues of each paralogue are also highest for MSMB3. Orange: mouse Msmb shows higher identity to MSMB2 than to either MSMB1 or MSMB3. [file 1471-2148-11-373-S4.PDF]

% identity \ % similarity		mouse Msmb	MSMB1				MSMB2			MSMB3			
			ostrich	chicken	turkey	zebra finch	chicken	turkey	zebra finch	duck	chicken	turkey	zebra finch
mouse Msmb			25	30	29	28	32	34	36	24	23	24	25
MSMB1	ostrich	35		52	49	54	31	30	35	32	34	33	29
	chicken	43	66		83	60	39	40	40	35	35	35	32
	turkey	43	63	90		57	37	37	37	32	32	32	30
	zebra finch	42	62	69	66		39	39	43	35	36	35	30
MSMB2	chicken	47	42	50	50	53		89	56	27	28	27	25
	turkey	50	42	50	50	51	96		53	27	28	27	25
	zebra finch	53	44	52	51	55	70	68		30	29	28	25
MSMB3	duck	41	42	52	48	49	44	44	45		91	91	82
	chicken	41	43	51	48	50	44	44	45	94		98	81
	turkey	42	43	52	49	49	44	44	44	95	99		81
	zebra finch	43	41	51	46	47	43	43	47	92	90	91	
